# A Quantitative Real-Time RT-PCR Assay for the Detection of* Venezuelan equine encephalitis virus* Utilizing a Universal Alphavirus Control RNA

**DOI:** 10.1155/2016/8543204

**Published:** 2016-11-29

**Authors:** Ariel Vina-Rodriguez, Martin Eiden, Markus Keller, Winfried Hinrichs, Martin H. Groschup

**Affiliations:** ^1^Institute for Novel and Emerging Infectious Diseases, Friedrich-Loeffler-Institut, Greifswald, Insel Riems, Germany; ^2^Department of Molecular Structural Biology, Institute for Biochemistry, University of Greifswald, Greifswald, Germany

## Abstract

*Venezuelan equine encephalitis virus* (VEEV) is an* Alphavirus* from the family* Togaviridae* that causes epizootic outbreaks in equids and humans in Central and South America. So far, most studies use conventional reverse transcriptase PCR assays for the detection of the different VEEV subtypes. Here we describe the development of a TaqMan quantitative real-time reverse transcriptase PCR assay for the specific detection and quantitation of all VEEV subtypes which uses in parallel a universal equine encephalitis virus control RNA carrying target sequences of the three equine encephalitis viruses. The control RNA was used to generate standard curves for the calculation of copy numbers of viral genome of* Eastern equine encephalitis virus* (EEEV),* Western equine encephalitis virus* (WEEV), and VEEV. The new assay provides a reliable high-throughput method for the detection and quantitation of VEEV RNA in clinical and field samples and allows a rapid differentiation from potentially cocirculating EEEV and WEEV strains. The capability to detect all known VEEV variants was experimentally demonstrated and makes this assay suitable especially for the surveillance of VEEV.

## 1. Introduction

*Western equine encephalitis virus* (WEEV),* Eastern equine encephalitis virus* (EEEV), and* Venezuelan equine encephalitis virus* (VEEV) are arthropod-borne (arbo) viruses of the genus* Alphavirus* of the virus family* Togaviridae*. To date these viruses are restricted to the Americas but due to worldwide travelling and trade they might be introduced also to other parts of the world in the future. All three equine encephalitis viruses are classified as Category B agent by the Centers for Disease Control and Prevention, Atlanta (https://emergency.cdc.gov/agent/agentlist-category.asp). They are transmitted by sanguivorous mosquitoes within bird (WEEV, EEEV, and epizootic VEEV (epizootic strains)) or rodent populations (VEEV, enzootic strains), respectively. Infections in reservoir hosts do not lead to obvious clinical signs. However, severe diseases can occur when equines and humans are infected with epizootic subtypes by biting mosquitoes. In the last decade 662 equine cases with 302 fatalities of VEE were reported to the OIE and 75,000–100,000 human cases with more than 300 fatalities were counted in the most recent outbreak in Venezuela and Colombia in 1995 [1]. For some epizootic VEEV strains a productive replication followed by successful intra- and interspecies transmission cycles was observed in horses and humans [2–6].

In the time period 2007 to 2012 a total of 1926 EEEV and 3 WEEV associated equine cases were diagnosed in the United States (http://www.oie.int/). In the time period 1964 to 2009 the CDC registered 639 human WEEV and 260 EEEV cases with only few fatalities. It was shown recently in an experimental animal model that all three viruses are transmissible by aerosols [7–9].

In general, PCR-diagnostic for emerging viruses follows different questions: for the surveillance during an epidemic or the confirmation of infections from a known source a PCR specific for some virus variant may be sufficient. In contrast to prevent the introduction of the virus into a corresponding region or country, the use of an assay with an experimentally proven capability is necessary to detect every known virus variant. According to this several conventional RT-PCRs were developed to qualitatively detect VEEV, EEEV, and WEEV genome sequences and real-time reverse transcriptase PCRs (RT-qPCR) for EEEV and WEEV were published [10–13]. However, no RT-qPCR assay was available at the date of our study for the specific detection of different VEEV subtypes. A VEEV diagnosis is presently confirmed mostly by conventional RT-PCR using broad-range primer pairs covering the whole genus* Alphavirus* followed by subsequent amplicon sequencing [14]. Recent publications experimentally demonstrated RT-qPCR assays for detection of the VEEV vaccine strain TC-83 but without proven experimental demonstration of the assay's sensitivity and efficiency regarding other VEEV subtypes [15, 16]. In this study we are introducing a general purpose, rapid, one-step quantitative RT-qPCR assay for the sensitive and specific detection of all VEEV subtypes in combination with an internal calibrator construct which in turn can be used in the quantification of the three equine encephalitis viruses.

## 2. Materials and Methods

### 2.1. Primer Design

Multiple sequence alignments of VEEV sequences were performed using Vector NTI Advanced v.10 (Invitrogen, Carlsbad, CA, USA) and MEGA Software [17] to reveal primers, as well as a probe. For this purpose, a total of 33 VEEV sequences were retrieved from the GenBank database. Published broad-range primers, which target the nsP1 region of Alphaviruses and previously used within a conventional RT-PCR protocol [14], were modified by the insertion of a degenerated base in each of the forward and the reverse primer and complemented with a FAM- (6-carboxyfluorescein-) labelled probe, which specifically targets VEEV sequences (Table 1) and enables the application of a quantitative real-time RT-PCR protocol.

### 2.2. Quantitative Real-Time RT-PCR (RT-qPCR)

RT-qPCR was carried out by using a commercial kit (QuantiTect RT-PCR kit, Qiagen, Germany). After the reverse transcription (50°C for 30 minutes) the DNA was denatured (95°C for 15 min). Amplification cycles included denaturation (95°C for 15 sec), annealing (55°C for 30 sec), and elongation (72°C for 30 sec) steps. Ct values were determined by the CFX96 software (Bio-Rad, USA).

### 2.3. Synthetic Calibrator

**Figure 1:**
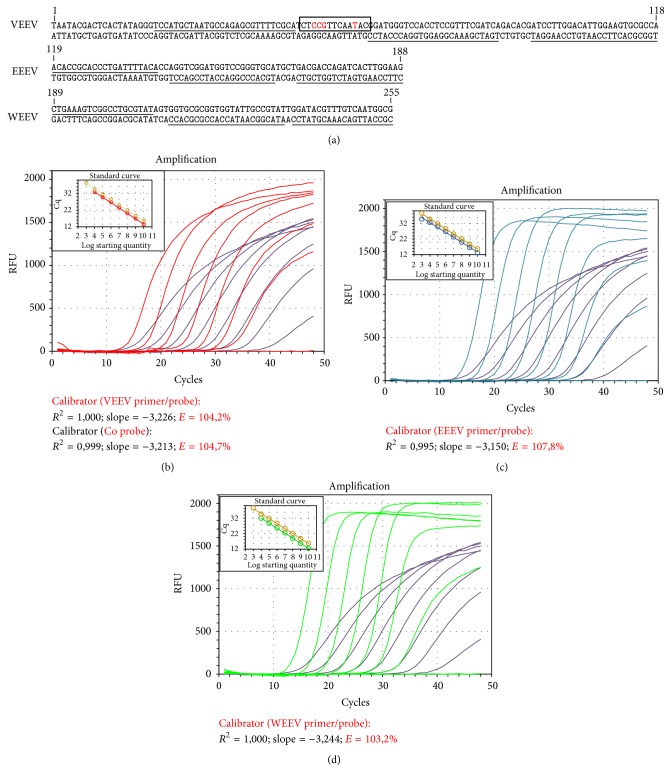
The nucleotide sequence of the synthetic construct used for calibration of the EEV-specific qRT-PCRs (a). The target sequences (underlined) for the specific qRT-PCRs are cloned into the vector pCR2.1-TOPO. Within the VEEV target region a modified sequence (framed) allows the differentiation of the synthetic calibrator from viral sequences. Nucleotide exchanges are indicated in red. Amplification curves of the qRT-PCRs specific for VEEV (b), EEEV (c), and WEEV (d) using the synthetic calibrator template. Amplification curve of the synthetic calibrator targeted with the control probe is indicated in olive, respectively. Standard curves (see enclosed boxed figures) were obtained by Ct values plotted against the log of the starting quantity. Calculated correlation coefficients (*R* ^2^), slopes, and amplification efficiencies (*E*) are depicted below the corresponding figures.

To determine the copy number of viral genomes a synthetic calibrator was developed, which comprises a T7 RNA polymerase promoter and the target sequences for the RT-qPCRs of EEEV, WEEV, and VEEV (Figure 1(a)) cloned into the pCR2.1 vector (Eurofins MWG Operon, Germany). The EEEV and WEEV sequences include targets for primer and probes adopted unmodified from the literature [10] (Table 1), but the corresponding probe target sequences were placed on the complementary strand in order to generate a unique (different) amplicon sequence, discriminable from the original virus sequence yet maintaining the same nucleotide composition. In addition, within the VEEV target region the original virus sequence 5′-CTGGCTTCAAAAC-3′ was changed to 5′-CTCCGTTCAATAC-3′ in order to discriminate unambiguously the synthetic RNA from viral RNA and to exclude false positive signals in samples potentially contaminated with synthetic RNA. This specific synthetic RNA sequence section can be detected only by a control probe (Table 1, VEEV-Coprobe). The plasmid was linearized with* Xba*I and subsequently transcribed into RNA and the DNA degraded using the Riboprobe® Combination System, T3/T7 RNA Polymerase (Promega Corporation's, Madison, WI, USA), and the QIAamp Viral RNA Mini Kit (Qiagen) was used for RNA isolation (without carrier RNA). The RNA concentration was estimated with the Quant-It™ RNA Assay Kit, Broad-Range (Invitrogen). The copy number of the synthetic RNA was calculated from the RNA concentration and the molecular mass of the RNA transcript.

## 3. Results and Discussion

**Figure 2:**
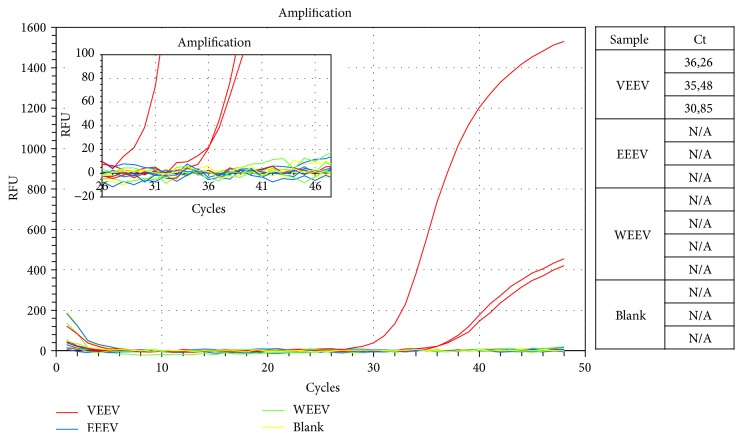
Specificity of the VEEV specific primer-probe combination. Specific amplification of VEEV derived RNA (red) by qRT-PCR. No amplification of EEEV (blue) and WEEV (green) derived RNA. Insert shows the boxed region at higher magnification.

In the here presented study, we are introducing a rapid, sensitive, and reliable one-step quantitative RT-PCR assay for VEEV as well as a synthetic RNA construct which can be used as calibrator for the quantification of alpha virus associated equine encephalitis viruses. PCR was carried out on serial dilutions of the synthetic RNA in a one-step RT-qPCR and Ct values were eventually plotted proportionally to the logarithm of the input copy numbers to produce standard quantitation curves. Negative controls were included in each run. The synthetic RNA was concurrently amplified using primer and probes for VEEV, WEEV, and EEEV, respectively (Figures 1(b)–1(d)). In addition the synthetic calibrator was amplified with primer for VEEV and detected by the VEEV-Coprobe. Both assays run independently in a single-plex format. All four standard curves exhibit a correlation coefficient >99% and an amplification efficiency of about 103–107% over a linear range of 10^2^ to 10^10^ copies. Based on the corresponding standard curves the sensitivity and viral load for different EEV strains in concurrent runs could be determined: the limit of detection (LOD) corresponded to 66,2 copies per *μ*L (Ct = 37,24) for VEEV (TC-83 strain), at 736 copies (Ct = 32,38) for WEEV (McMillan strain), and at 316 copies per *μ*L (Ct = 34,21) for EEEV (New Jersey strain) (Table 2). Since standard curves form part of every run, the copy number from each analysed sample can be determined. Therefore samples with copy numbers above 1 copy per *μ*L (the theoretical detection limit) are considered to be positive. VEEV specific primer/probe combination did not detect other equine encephalomyelitis viruses (EEEV, WEEV) (Figure 2) or closely related species (Chikungunya virus, Sindbis virus, and Ross River virus; data not shown).

**Figure 3:**
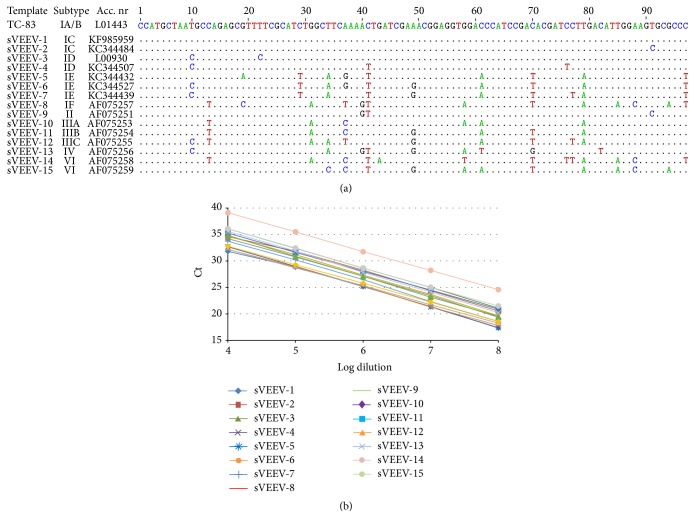
Comparison of the consensus sequences of different VEEV subtypes. (a) Sequences of synthetic RNA constructs (sVEEV) encompass the target region of the VEEV specific qRT-PCR. Nucleotides with mismatch to the reference sequence are indicated. (b) Standard curves of serial diluted sVEEV were obtained by Ct values plotted against the log of diluted template.

To further assess the performance of the VEEV specific RT-qPCR we used 15 synthetic RNA constructs (sVEEV) encompassing the target region and representing 10 different VEEV subtypes (Figure 3(a)). This included all combinations of observed mutations in the primers and probe target regions. sVEEV were designed as oligonucleotides with a 5′ T7 RNA polymerase promoter sequence and were transcribed* in vitro* as aforementioned. All VEEV subtypes were successfully detected by the novel RT-qPCR assay with a suitable sensitivity and high performance as demonstrated by linear standard curves over 5 logs (Figure 3(b)). *R* ^2^ values and slope indicate good precision and high efficiency (Table 3). To evaluate the effect of nucleotide changes to the PCR amplification efficiency we applied the relative threshold cycle (RTC) method, which refers to mean Ct-differences (mean ΔCt) of the corresponding sVEEV template compared to the unmodified template [18]. The data indicate that most nucleotide changes exhibited only small or moderate reduction of the RTC efficiency. Only sVEEV-16, representing one variant of subtype VI, showed a stronger decline in RTC efficiency which is probably caused by 13 nucleotide exchanges compared to the reference template. In summary this assay can be used whenever a sensitive and high-throughput detection or quantification of VEEV RNA is needed, for example, for confirmation of virus presence in patients, during infection experiments or large screening of field probes. But it is particularly useful when a proven application for the detection of all known VEEV variants is required, for example, to prevent the introduction of any virus variant into a so far virus free region or country.

## 4. Conclusions

We report here the first experimental evidence of a quantitative real-time RT-PCR assay for the sensitive and specific detection of all known VEEV subtypes or sequence variants. The synthetic calibrator RNA allows the determination of viral genome equivalents of VEEV as well as WEEV and EEEV by a one-step RT-qPCR reaction.

## Figures and Tables

**Table 1:** Primers and probes selected for equine encephalitis virus-specific quantitative reverse transcription polymerase chain reaction.

Target	Primer or probe	Sequence (5′ → 3′)	Genome position	Reference
*Eastern equine encephalomyelitis virus* (EEEV)	EEE9391	ACACCGCACCCTGATTTTACA	9391–9411 (s)	
	EEE9459c	CTTCCAAGTGACCTGGTCGTC	9459–9439 (as)	[10]
	EEE.9414probe	FAM-TGCACCCGGACCATCCGACCT-TAMRA	9414–9434 (s)	

*Western equine encephalomyelitis virus* (WEEV)	WEE10,248	CTGAAAGTCGGCCTGCGTAT	10,248–10,267 (s)	
	WEE 10,314c	CGCCATTGACGAACGTATCC	10,314–10,295 (as)	[10]
	WEE 10,271probe	FAM-ATACGGCAATACCACCGCGCACC-TAMRA	10,271–10,293 (s)	

*Venezuelan equine encephalomyelitis virus* (VEEV) and synthetic calibrator	AlphaVIR966F	TCCATGCTAATGC*Y*AGAGCGTTTTCGCA	151–178 (s)	Modified [14]
	AlphaVIR966R	TGGCGCACTTCCAATGTC*H*AGGAT	248–225 (as)	
	*INEID-VEEV probe*	FAM-*TGATCGARACGGAGGTRGAMCCATCC*-TAMRA	193–218 (s)	This study
	*VEEV-Coprobe*	VIC-*CTCCGTTCAATAC*-MGB-NFQ^*∗*^	180–192 (s)	This study

The synthetic calibrator RNA is specifically detected by the VEEV-Coprobe in combination with the AlphaVIR966F and AlphaVIR966R primers. Y, H, R, and M are designed for degenerative bases, where Y = C/T, H = A/C/T, R = A/G, and M = A/C. Modifications compared to the original sequence as well as novel sequences were indicated in italic font. ^*∗*^MGB: minor groove binder; NFQ: Nonfluorescent quencher.

**Table 2:** Sensitivity of RT-qPCR assays for VEEV, WEEV, and EEEV strains and determination of copy number.

RNA	Dilution	Ct	Copies/*µ*L
VEEV	10^−2^	27,94	70200
	10^−3^	30,69	8840
	10^−4^	33,75	894
	10^−5^	35,51	240
	10^−6^	37,24	66
	10^−7^	no Ct	0

WEEV	10^−2^	21,76	2880000
	10^−3^	25,13	216000
	10^−4^	28,64	13780
	10^−5^	31,1	2060
	10^−6^	32,38	736
	10^−7^	no Ct	0

EEEV	10^−3^	29,67	12760
	10^−4^	32,55	1212
	10^−5^	34,22	316
	10^−6^	no Ct	0
	10^−7^	no Ct	0

**Table 3:** Relative threshold cycle (RTC) amplification efficiencies of synthetic VEEV (sVEEV) RNA constructs.

Template	sVEEV −1	sVEEV −2	sVEEV −3	sVEEV −4	sVEEV −5	sVEEV −6	sVEEV −7	sVEEV −8	sVEEV −9	sVEEV −10	sVEEV −11	sVEEV −12	sVEEV −13	sVEEV −14	sVEEV −15
Dilution	Ct	Ct	Ct	Ct	Ct	Ct	Ct	Ct	Ct	Ct	Ct	Ct	Ct	Ct	Ct

10^−4^	17,3	20,6	19,4	19,6	17,4	21,1	18,6	17,8	18,3	18,5	20,9	19,6	20,2	24,6	21,5
10^−5^	21,4	24,4	23,4	23,7	21,3	25,0	22,3	21,3	21,7	22,2	24,5	23,1	24,1	28,2	25,0
10^−6^	25,2	28,3	27,1	27,3	25,2	28,7	26,5	25,3	25,4	25,8	28,0	27,1	27,8	31,7	28,6
10^−7^	28,9	31,8	30,8	31,2	29,0	32,4	30,2	28,9	28,7	29,3	31,6	30,9	31,9	35,5	32,4
10^−8^	31,8	34,6	34,2	34,7	32,7	36,1	33,7	32,2	32,2	32,8	35,3	34,9	35,6	39,1	36,1

Slope |*R* ^2^	−2,99| 0,9969	−2,91| 0,9959	−3,05| 0,9992	−3,1| 0,9995	−3,16| 0,9998	−3,08| 0,9998	−3,16| 0,9992	−2,98| 0,9993	−2,85| 0,9999	−2,92| 0,9999	−2,95| 1	−3,17| 0,9997	−3,18| 0,9999	−2,99| 0,9999	−3,00| 0,9998

Mean ΔCt	**0,0**	**−3,0**	**−2,1**	**−2,4**	**−0,2**	**−3,7**	**−1,4**	**−0,2**	**−0,3**	**−0,8**	**−3,2**	**−2,2**	**−3,0**	**−6,9**	**−3,8**
Mean RTC	**1,0**	**0,12**	**0,24**	**0,19**	**0,87**	**0,08**	**0,39**	**0,89**	**0,80**	**0,57**	**0,11**	**0,22**	**0,12**	**0,01**	**0,07**

ΔCt is calculated as mean difference of corresponding Ct values compared to unmodified reference template sVEEV-1 across all template dilutions. RTC is calculated according RTC = 2^Δct^.
